# Machine learning analyses identify multi-modal frailty factors that selectively discriminate four cohorts in the Alzheimer’s disease spectrum: a COMPASS-ND study

**DOI:** 10.1186/s12877-023-04546-1

**Published:** 2023-12-11

**Authors:** Linzy Bohn, Shannon M. Drouin, G. Peggy McFall, Darryl B. Rolfson, Melissa K. Andrew, Roger A. Dixon

**Affiliations:** 1 Department of Psychology, University of Alberta, P217 Biological Sciences Building, Edmonton, AB T6G 2E9 Canada; 2 Neuroscience and Mental Health Institute, University of Alberta, 2-132 Li Ka Shing Center for Health Research Innovation, Edmonton, AB T6G 2E1 Canada; 3 Department of Medicine, Division of Geriatric Medicine, University of Alberta, 13-135 Clinical Sciences Building, Edmonton, AB T6G 2G3 Canada; 4 Department of Medicine, Division of Geriatric Medicine, Dalhousie University, 5955 Veterans’ Memorial Lane, Halifax, NS B3H 2E1 Canada

**Keywords:** Frailty, Deficit accumulation, Subjective cognitive impairment, Mild cognitive impairment, Alzheimer’s disease, Canadian Consortium on Neurodegeneration in Aging, Comprehensive Assessment of Neurodegeneration and Dementia, Random forest analysis, Tree Shapley additive exPlanation values, Machine learning

## Abstract

**Background:**

Frailty indicators can operate in dynamic amalgamations of disease conditions, clinical symptoms, biomarkers, medical signals, cognitive characteristics, and even health beliefs and practices. This study is the first to evaluate which, among these multiple frailty-related indicators, are important and differential predictors of clinical cohorts that represent progression along an Alzheimer’s disease (AD) spectrum. We applied machine-learning technology to such indicators in order to identify the leading predictors of three AD spectrum cohorts; viz., subjective cognitive impairment (SCI), mild cognitive impairment (MCI), and AD. The common benchmark was a cohort of cognitively unimpaired (CU) older adults.

**Methods:**

The four cohorts were from the cross-sectional Comprehensive Assessment of Neurodegeneration and Dementia dataset. We used random forest analysis (Python 3.7) to simultaneously test the relative importance of 83 multi-modal frailty indicators in discriminating the cohorts. We performed an explainable artificial intelligence method (Tree Shapley Additive exPlanation values) for deep interpretation of prediction effects.

**Results:**

We observed strong concurrent prediction results, with clusters varying across cohorts. The SCI model demonstrated excellent prediction accuracy (AUC = 0.89). Three leading predictors were poorer quality of life ([QoL]; memory), abnormal lymphocyte count, and abnormal neutrophil count. The MCI model demonstrated a similarly high AUC (0.88). Five leading predictors were poorer QoL (memory, leisure), male sex, abnormal lymphocyte count, and poorer self-rated eyesight. The AD model demonstrated outstanding prediction accuracy (AUC = 0.98). Ten leading predictors were poorer QoL (memory), reduced olfaction, male sex, increased dependence in activities of daily living (*n* = 6), and poorer visual contrast.

**Conclusions:**

Both convergent and cohort-specific frailty factors discriminated the AD spectrum cohorts. Convergence was observed as all cohorts were marked by lower quality of life (memory), supporting recent research and clinical attention to subjective experiences of memory aging and their potentially broad ramifications. Diversity was displayed in that, of the 14 leading predictors extracted across models, 11 were selectively sensitive to one cohort. A morbidity intensity trend was indicated by an increasing number and diversity of predictors corresponding to clinical severity, especially in AD. Knowledge of differential deficit predictors across AD clinical cohorts may promote precision interventions.

**Supplementary Information:**

The online version contains supplementary material available at 10.1186/s12877-023-04546-1.

## Background

Frailty can be characterized by the presence of multiple constituent factors representing dynamic and heterogeneous combinations of geriatric conditions, clinical symptoms, biomarkers, medical signals, cognitive characteristics, and even health beliefs and practices. A leading and productive approach to evaluating frailty involves calculating a continuous index that reflects the number of such deficits acquired relative to the total number considered [1]. Emerging evidence from separate studies indicates that higher frailty levels are linearly (and positively) associated with subjective cognitive impairment (SCI) [2, 3], mild cognitive impairment (MCI) [4], and Alzheimer’s disease (AD) [5, 6]. Accordingly, we reasoned that frailty represents a promising framework for identifying the key factors that predict geriatric conditions representing progression along an AD spectrum.

Available studies evaluating frailty and clinical risk for SCI, MCI, or AD have successfully used overall summary frailty scores (such as indices or phenotypes, varying primarily in level or magnitude), without specific attention to the number of indicators or breadth of modalities represented across the predictors. However, several recent reviews have expressed the importance for studies to investigate whether there are specific frailty indicators or combinations thereof that are differentially associated with AD and related neurodegenerative disorders [7, 8]. In particular, it may be profitable to apply integrated, data-driven neuroinformatics approaches [7, 9–12] to exploring whether and which subsets of frailty factors might optimize differential prediction of a spectrum of geriatric and impairment conditions.

The present study builds on the foundation of a leading frailty research tradition that emphasizes the importance of comprehensive collections of frailty-related factors [13, 14] but focuses on identifying salient indicators that differentially distinguish cognitively unimpaired (CU; or asymptomatic) groups from those occupying clinical positions along a continuum of impairment and dementia. The overall objective was to determine which frailty indicators discriminate a CU cohort from SCI, MCI, and AD cohorts using computationally competitive machine learning (ML) classifier analyses. Individuals classified as having SCI report subjective cognitive complaints or concerns in the absence of objective signs of impairment in measured aspects of cognitive function [15]. Although phenotypically similar to CU aging, this condition may represent an early and potentially modifiable risk phase for exacerbated objective cognitive decline [10, 16], as well as, successively, MCI [17] and AD [18]. Among the progressive clinical differences between CU, SCI, MCI, and AD cohorts are accumulation of multiple modalities of AD risk factors and symptoms [19, 20], as well as increased severity of functional and cognitive impairment owing to accumulating AD neuropathology [21, 22]. Accordingly, specific hypotheses for this study included cohort differences in (a) detected clusters of important predictors, (b) accuracy of prediction models, and (c) clinical intensity of important frailty indicators.

Recently, ML classifier approaches have been applied to modeling large-scale and multi-modal indicators of a range of morbidities and perturbations related to various biological, medical, and other risk domains that negatively influence brain and cognitive health [11, 23, 24], progression toward neurodegeneration [12, 25, 26], and even characteristics of brain resilience to AD-related adversities [27–29]. Such approaches have also been applied to multi-faceted frailty-related indicators sampled from CU older adults [30–32] and selected geriatric clinical cohorts [33–35], though none specifically focused on discriminating clinical conditions representing the full AD spectrum. Accordingly, the present study is the first to our knowledge to determine which indicators, from a multi-modal roster of frailty-related deficits and risk factors, discriminate CU older adults from those with SCI, MCI, or mild AD. The findings have applications to detecting novel precision targets for early intervention or treatment protocols that produce positive downstream effects on offsetting or delaying the incidence or progression of SCI, MCI, or even AD.

We used newly available data from the Canadian Consortium on Neurodegeneration in Aging (CCNA). The Comprehensive Assessment of Neurodegeneration and Dementia (COMPASS-ND) dataset features well-characterized cohorts of aging and neurodegeneration, including CU, three groups associated with the AD clinical spectrum, and a large roster of frailty-related indicators [36, 37]. We specified three research goals (RG) corresponding to a series of three ML-based random forest (RF) classifier analyses designed to identify the most important factors that discriminate the benchmark group (CU) from SCI (RG1), MCI (RG2), and AD (RG3) cohorts. Accordingly, we (a) simultaneously tested a comprehensive set of multi-modal morbidities, deficits, and risk characteristics and (b) compared and contrasted the important predictors of the three clinical cohorts. We then integrated an explainable artificial intelligence method— Tree Shapley Additive exPlanation values (Tree SHAP) [38]— which facilitated deep interpretation of the direction, prevalence, and magnitude of prediction effects generated by the black-box RF algorithm [39]. Such interpretation goals are particularly relevant in clinical aging and dementia research as they point toward potential precision targets for intervention [38]. In sum, this analytical approach was expected to reveal previously unknown signatures of elevated risk associated differentially with SCI, MCI, and AD clinical status.

## Methods

### Participants

A detailed methodological summary of the COMPASS-ND study has been previously published [36, 37]. Briefly, participants were recruited from 31 data collection sites across Canada, most of which were academic clinical-research settings. Ethics approval was obtained from the Research Ethics Committee or Institutional Review Board of each participating data collection site. All participants and their legal guardians provided written informed consent. Older adults with the following criteria were ineligible to participate in the COMPASS-ND study protocol [36, 37]: (a) presence of significant known chronic brain disease, multiple sclerosis, a serious developmental handicap, malignant tumors, Huntington’s disease, and other rarer brain illnesses; (b) ongoing drug or alcohol abuse; (c) total score < 13 on the Montreal Cognitive Assessment [40]; (d) symptomatic stroke within the previous year; and (e) unwilling or unable to undergo magnetic resonance imaging scan. Eligible study participants (a) were sufficiently proficient in English or French and (b) had a study partner with whom they interacted on a weekly basis. Clinical diagnostic status was determined by experienced clinicians involved in the COMPASS-ND study using current diagnostic criteria [36]. For the current study, specific exclusionary criteria were a diagnosis of subcortical ischemic vascular MCI, dementia of mixed etiology, frontotemporal dementia, Parkinson’s disease, and Lewy body dementia. We assembled data for deeply phenotyped participants representing the following cohorts: CU (*n* = 60), SCI (*n* = 36), MCI (*n* = 116), and AD (*n* = 43). Descriptive statistics for the final sample are presented in Table 1 (*N* = 255; *M*age = 71.18, *SD* = 6.81; 58% female; 92% non-Hispanic White).

**Table 1:** Demographic and Clinical Characteristics Disaggregated by Cohort

Characteristic	CU (n = 60)	SCI (n = 36)	MCI (n = 116)	AD (n = 43)	sig
n (% female)	49 (82%)^a^	30 (83%)^a^	57 (49%)^b^	13 (30%)^c^	^***^
Age (years)	69.23 (5.52)^a^	69.62 (6.81)^a^	71.16 (6.48)^a^	75.26 (7.70)^b^	^***^
Education (years)	15.84 (3.15)	17.49 (3.11)	15.75 (3.89)	15.34 (4.37)	^*ns*^
n (% married)	37 (62%)^a^	17 (47%)^a^	75 (65%)^a^	35 (81%)^b^	^*^
n (% non-Hispanic White)	58 (97%)^a^	34 (94%)^a,b^	100 (86%)^b^	42 (98%)^a^	^*^
n (% community dwelling)^^^	60 (100%)	36 (100%)	116 (100%)	43 (100%)	^*ns*^
n (% residing with spouse)^^^^	43 (72%)^a,b^	20 (56%)^b^	85 (73%)^a^	37 (86%)^a^	^*^
MoCA	27.90 (1.50)^a^	27.81 (1.33)^a^	24.28 (3.08)^b^	18.63 (3.56)^c^	^***^

Results are presented as mean (standard deviation) unless noted as otherwise. *p*-values are based on one-way analysis of variance (with post-hoc Tukey tests) or chi-square tests, as appropriate. ^^^ Self-reported as living in a house, apartment, or condo. ^^^^ Self-reported as living with a spouse or significant other. ^a,b,c^ Denotes values that differ significantly. Abbreviations: CU, cognitively unimpaired; SCI, subjective cognitive impairment; MCI, mild cognitive impairment; AD, Alzheimer’s disease; sig, significance; *ns*, not significant; MoCA, Montreal Cognitive Assessment.

^*^p-value < 0.05 ^***^p-value < 0.001.

### Measures

#### Multi-modal frailty-related indicators

We began by assembling 102 frailty-related indicators of morbidity, deficits, and risk factors from the COMPASS-ND database. They were determined to represent the following 17 frailty-related domains: instrumental activities of daily living (ADL), basic ADL, physical activity, mobility, QoL, anthropometric measures, sensory function, sleep, functional indicators, exhaustion, self-reported health, cardiorespiratory health, clinical symptoms or diseases, emotional well-being, oral health and nutritional factors, fluid biomarkers, and sex. Procedures for collecting these data from study participants included self-report, physical examinations, and formal tests with standardized scales. Binary indicators were coded as 0 (deficit absent) or 1 (deficit present). Continuous indicators were coded such that values ranged between 0 (no deficit recorded) and 1 (deficit is maximally expressed) [1]. The only exception to this practice was the polypharmacy variable, which ranged between 0 (0–4 medications) and 2 (14+ medications) [41, 42]. Where applicable, cut points for continuous indicators were provided in the COMPASS-ND database or derived from previous empirical research. We note that some of the present indicators were used in an earlier study to operationalize a continuous index and examine frailty prevalence across several clinical cohorts in the COMPASS-ND database [41].

#### Fluid biomarkers

Biosamples of blood, saliva, and urine were collected using established operating procedures [36]. We assembled data for 67 fluid biomarkers that were dichotomized in the COMPASS-ND database as 0 (within established reference range) or 1 (outside established reference range) [43, 44].

#### Screening prospective frailty-related indicators and fluid biomarkers

We screened the prospective frailty-related indicators and fluid biomarkers for eligibility for inclusion in two standard pre-processing steps. First, we assembled three separate datasets (one for each pairwise RF comparison) and removed indicators with a rate of missingness > 50%. This criterion cut-off accords with previous ML [45–47] and frailty research [34]. Second, we removed categorical indicators in which < 10% of participants in each cohort were recorded as having the associated deficit (i.e., noisy features). Removing such indicators increases learning accuracy, facilitates model interpretation, and decreases running time [35, 48]. The final number of indicators submitted to RF analysis was 64 for SCI, 65 for MCI, and 75 for AD (total *n* of individual indicators across cohorts = 83). For the SCI model, the average percentage of missing data was 2% for binary predictors and 0.4% for continuous predictors. For the MCI model, the average percentage of missing data was 2% for binary predictors and 0.8% for continuous predictors. For the AD model, the average percentage of missing data was 4% for binary predictors and 0.7% for continuous predictors. The average percentage of missing data (i.e., collapsing across binary and continuous predictors) was 1% in both the SCI and MCI model, and 2% in the AD model. We present the 83 frailty-related indicators (disaggregated by domain) together with the corresponding response scales in Supplement Table 1.

#### Sex

Biological sex was measured in a binary fashion with the available intake item requesting participants to self-report whether they are male or female.

### Analytical approach

The approach involved two integrated analytic steps for each RG; viz., ML identification of the leading discriminating factors and explainable artificial intelligence method (Tree SHAP) for deep interpretation of prediction effects.

#### Step 1: Machine learning predictor analyses

The relative predictive importance of the evaluated indicators in discriminating CU from SCI, MCI, or AD was accomplished with RF classifier analysis [49]. Analyses were performed using Python (3.8) [50] and the scikit-learn package (*RandomForestClassifier*) [51] with the following hyperparameters: *n*_estimators = 1000 [24, 52–54], max_depth = 3, max_features = auto [39]. RF analysis is a recursive partitioning method, meaning that it combines predictions across multiple classification and regression trees, each of which is based on a random subset of participants (*n*) and predictors (*p*) [55]. This approach has several advantages relevant to the present clinical aging data. First, it has demonstrated utility for exploring high dimensional (i.e., *n* < *p*) [23, 24, 56] and mixed-type datasets (i.e., binary and continuous predictors) [23, 24], as well as discriminating selected AD-related clinical cohorts [11, 25, 57]. Second, it can examine many predictors simultaneously (including the possibility of non-linear factor dependencies and complex interactions) and returns a model with high prediction accuracy [39, 55]. Third, it is known to be robust to overfitting [58, 59], even in studies characterized by small and/or unbalanced (uneven) subsamples [11, 53, 60, 61], as in the present study. Third, descriptive variable importance measures that reflect the impact of each predictor on cognitive outcomes can be extracted [39, 62, 63].

RF models were evaluated using stratified *k*-fold cross-validation. This approach is recommended when the subsamples comprising the pairwise comparison are small and/or unbalanced [39]. These models ensure that each *k-*fold (or subsample) contains roughly the same proportion of each cohort as is represented in the overall sample. Specifically, we used stratified five-fold cross-validation to divide each pairwise dataset into five equally sized folds. Four of the five folds were used for training and the remaining fold was used for testing [64]. This process was repeated five times, until each fold had been used once for testing. The model then returned the following evaluation metrics, which were averaged across the cross-validation folds. The first and main metric was the *area under the receiver operating characteristic curve* (AUC), a summary measure of the model’s ability to distinguish between CU and the targeted clinical cohorts. AUC is interpreted such that 0.5 represents chance, 0.5–0.69 represents poor discrimination, 0.7–0.79 represents acceptable discrimination, 0.8–0.89 represents excellent discrimination, and **≥** 0.9 represents outstanding discrimination [65]. The second metric was *accuracy*, which refers to the total percentage of participants who were correctly classified as either CU or as belonging to the targeted clinical cohort (i.e., the fraction of true positives and true negatives over all model classifications). The third metric was *precision*, which represents the percentage of participants who were correctly classified into the target clinical cohort (calculated as true positives / (true positives + false positives)). The fourth metric was *sensitivity* (or *recall*), which reflects the percentage of participants from the target clinical cohort who were correctly classified as such (calculated as true positives / (true positives + false negatives)). The fifth metric was *F*_*1 *_*score*, an overall measure of model performance that represents a specific combination of precision and sensitivity. Technically, the F_1_ score is the “harmonic mean” (i.e., the reciprocal of the arithmetic mean) of precision and sensitivity. As such, it is calculated as 2 x (precision × sensitivity) / (precision + sensitivity). Values for the latter four metrics (i.e., accuracy, precision, sensitivity, F_1_ score) also ranged between 0 and 1, with higher values denoting better classification performance. In studies with unbalanced subsamples, AUC and F_1_ score are the most robust indicators of model performance [57]. We report these five metrics for each RF classification model but focus on AUC and F_1_ score when evaluating and interpreting (or assigning a qualitative label) to model fit.

Missing data were handled using *IterativeImputer* [66]— an advanced imputation approach derived from the Multiple Imputation by Chained Equations algorithm [66, 67]. *IterativeImputer* uses regularized linear regression to estimate (or predict) missing values as a function of all the other predictors in the model [51] and is an efficient and accurate imputation approach for mixed-type datasets [45]. Data were assumed to be either missing completely at random or missing at random [68]. We performed these analyses using the default *BayesianRidge* estimator and the *sklearn pipeline*. The *sklearn pipeline* allowed us to impute missing data (separately) within each cross-validation fold, thereby avoiding data leakage issues (i.e., between training and testing cross-validation folds) which can lead to overfitting, inflated model performance metrics, and reduced generalizability [69]. The *sklearn pipeline* involved the following two steps, which were conducted sequentially at each fold. First, missing data were imputed. Second, classification analyses were performed using RF analysis.

#### Step 2: Applying explainable artificial intelligence method for interpretation

Although RF analysis provides a robust and computationally competitive context from which to detect leading discriminative predictors, enhanced interpretations of the prediction patterns are afforded in this step. Specifically, we used Tree SHAP [25, 38, 70], which provides researchers with a unified framework for determining the relative importance (or model contribution) of the considered predictors. Briefly, Tree SHAP values are a robust analytical tool for (a) determining the relative magnitude of each predictor’s effect on model classifications while at the same time controlling for overfitting [25], (b) converging on a single unique solution that balances local accuracy, missingness, and consistency [38], and (c) drawing informed conclusions regarding the direction, magnitude, and prevalence of prediction effects.

RF analysis provides relative prediction values for all analyzed indicators. To aid in interpretation and comparison, we select the top 30 predictors in each RF model and display them in two Tree SHAP plots. We selected this number based on the consideration that the top 30 predictors in each analysis explained more than 80% of the model’s efficiency (or performance). The first figure displayed for each result is a Tree SHAP waterfall plot, which advances interpretation by (a) depicting the predictors in descending order of global importance (thus the rank ordering is consistent across figures) and (b) providing a visual representation of the RF analysis results [62]. Regarding the latter, this plot depicts the individual and cumulative ratio of the predictors’ contribution to the classification model (represented by the bars and curved line, respectively) [61]. The second figure for each result is a Tree SHAP summary plot. This figure advances interpretation in that (a) each dot represents an individual study participant; (b) the color of the dot represents the participant’s value on the associated predictor (red dot = high value, blue dot = low value); and (c) visual inspection of the location and distribution (or relative spread) of the colored dots along the x axis indicates the direction, prevalence, and magnitude of each predictor’s effect on the model output [38, 57, 62]. Direction of effect is indicated by the color of dots located to the right side of the vertical line on the x axis. Specifically, red dots to the right side of the vertical line on the x axis indicates that high values on the corresponding predictor increases risk for being classified into the targeted clinical cohort. Conversely, blue dots to the right side of the vertical line on the x axis indicates that low values on the corresponding predictor increases risk for classification into the targeted clinical cohort. Effect magnitude is indicated by the location of colored dots to the right side of the vertical line on the x axis. Specifically, dots located to the far side of the vertical line indicate that the corresponding predictor is associated with high magnitude effects, whereas dots clustered on the vertical line indicate that the corresponding predictor is associated with null effects. The prevalence of effect magnitudes is indicated by the distribution (or spread) of colored dots to the right side of the vertical line on the x axis. Specifically, clustered (or densely distributed) dots indicate the corresponding effect magnitude is highly prevalent in the study sample. Conversely, wide (or increasingly spread) dot distributions indicate the corresponding predictor is associated with lesser prevalent (or more varied) effect magnitudes.

## Results

### RG1: discriminating SCI and CU cohorts: machine learning analyses and Tree SHAP interpretation

Findings from the RF classifier analysis revealed that overall fit (or performance) of this model was excellent (AUC = 0.89; F_1_ score = 0.72), indicating reliable discrimination of these clinically neighbouring cohorts (see Table 2 for additional metrics).

**Table 2:** Random Forest Model Evaluation Metrics

RF Model	Indicators Tested	AUC	Accuracy	Precision	Recall	F_1_ Score
Discriminating SCI from CU	*n* = 64	0.89	0.79	0.94	0.58	0.72
Discriminating MCI from CU	*n* = 65	0.88	0.76	0.75	0.95	0.84
Discriminating AD from CU	*n* = 75	0.98	0.88	0.98	0.73	0.84

Evaluation metrics reflect average performance of the RF classification model across the five cross-validation folds. Each evaluation metric ranges between 0 and 1 (higher values denote better performance; see Methods section for further details). Abbreviations: RF, random forest; AUC, area under the receiver operating characteristic curve; SCI, subjective cognitive impairment; CU, cognitively unimpaired; MCI, mild cognitive impairment; AD, Alzheimer’s disease.

**Fig. 1:**
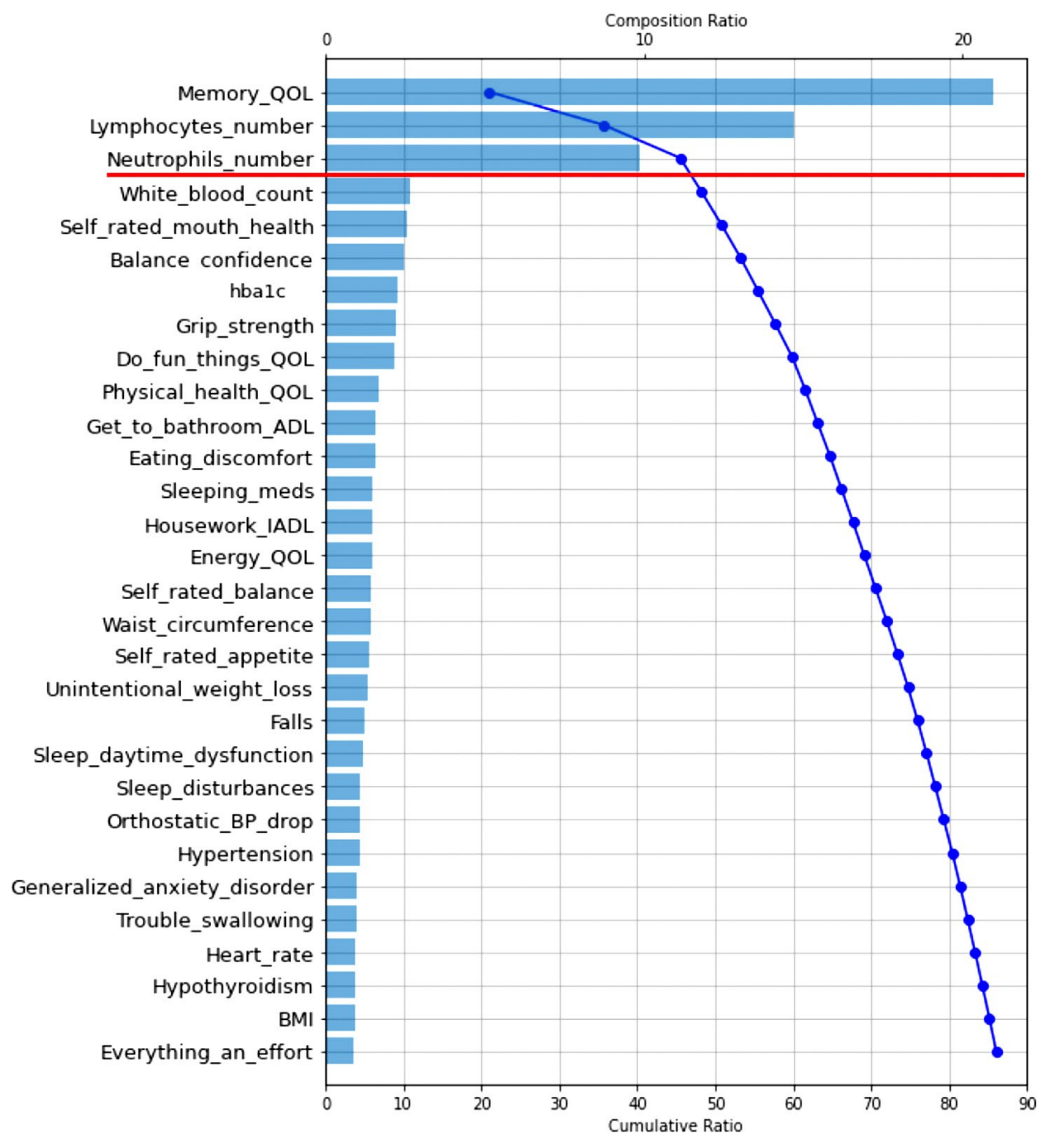
Tree SHAP waterfall plot depicting the top 30 predictors of subjective cognitive impairment. Predictors are plotted in descending order of model contribution. The bars depict the individual composition ratio (i.e., the amount that each predictor contributes to the classification model; see top of Figure for scale). The curved line represents the cumulative ratio (i.e., the total amount each successive predictor contributes to the model; see bottom of Figure for scale). Leading predictors are depicted above the red line. Abbreviations: QoL, quality of life; hba1c, glycated hemoglobin; ADL, basic activities of daily living; IADL, instrumental activities of daily living; meds, medication; BP, blood pressure; BMI, body mass index

As can be seen in the Tree SHAP waterfall plot (Fig. 1), the top three predictors are highlighted because (a) they have the highest global importance ratings (indexed by the composition ratio (i.e., blue bars; see top of Figure for scale and Figure legend for interpretation)), (b) there is an evident elbow (or break in the distribution) in the cumulative ratio at this cut-off, (c) they collectively explained approximately half (46%) of the model (indexed by the cumulative ratio (i.e., blue curved line; see bottom of Figure for scale and Figure legend for interpretation)), and (d) predictors below this cut-off contributed comparatively less to the classification model. The three leading predictors are QoL (memory; explained 21% of the cumulative ratio), lymphocyte count (explained 15%), and neutrophil count (explained 10%).

**Fig. 2:**
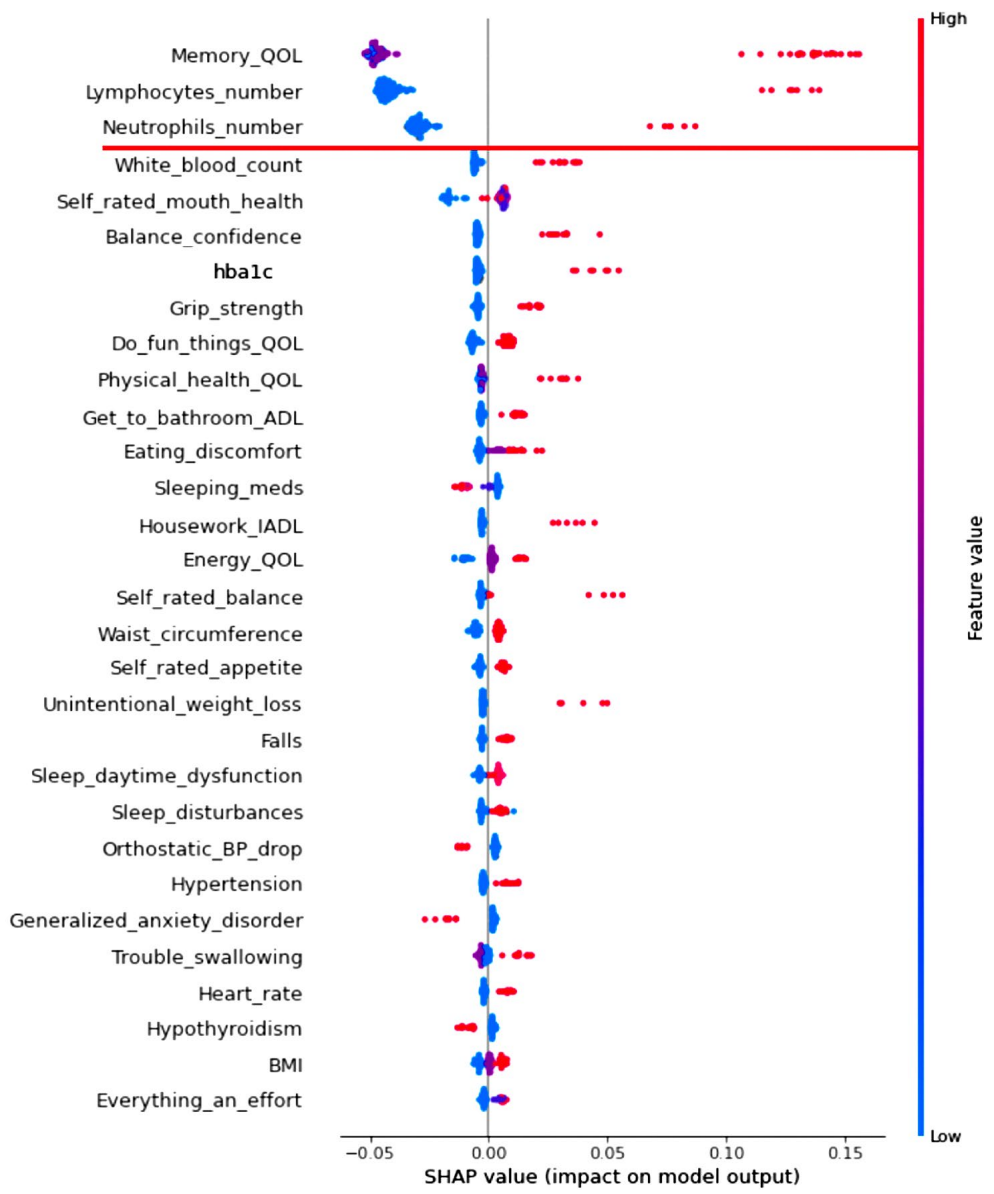
Tree SHAP summary plot depicting the top 30 predictors of subjective cognitive impairment. Predictors are plotted in descending order of model contribution. Each dot represents one study participant. These dots collectively represent the direction, prevalence, and magnitude of prediction effects (see Methods section for details). Leading predictors are denoted above the red line. Abbreviations: QoL, quality of life; hba1c, glycated hemoglobin; ADL, basic activities of daily living; IADL, instrumental activities of daily living; meds, medication; BP, blood pressure; BMI, body mass index

The Tree SHAP summary plot (Fig. 2) represents the direction, prevalence, and magnitude of effects associated with the top 30 predictors (response scales for each predictor are reported in Supplement Table 1). Regarding direction, for QoL (memory), red dots are located on the right side of the vertical line on the x axis, indicating that increasingly poor ratings elevate risk for SCI. Similarly, the observed patterns for lymphocytes and neutrophils show that abnormal counts increase risk for SCI. With respect to prevalence and effect magnitude, for QoL (memory), a large number of red dots are widely distributed along the far-right side of the vertical line on the x axis. This pattern indicates that, while the exact magnitude of effects varied across the considered cohorts, in general, poorer QoL (memory) is characterized by high magnitude effects. Similar findings were observed for abnormal lymphocyte count, with the exception that high magnitude effects were comparatively less prevalent in the overall study sample (as indicated by the smaller number of red dots on the far-right side of the vertical line). Regarding abnormal neutrophil count, effects of a more moderate magnitude were evidenced by a similar proportion of study participants.

### RG2: discriminating MCI and CU cohorts: machine learning analyses and Tree SHAP interpretation

**Fig. 3:**
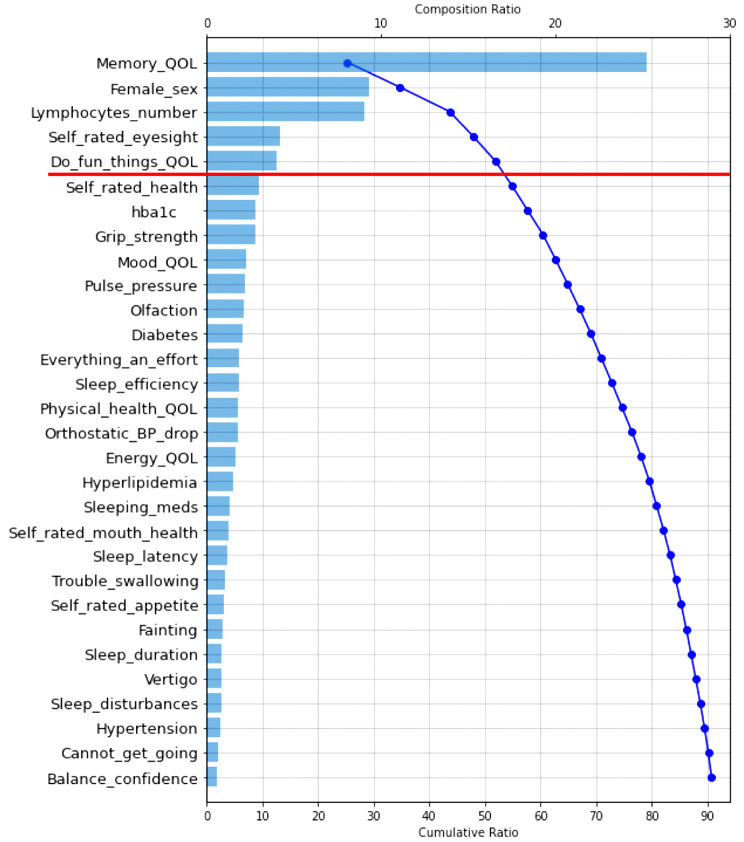
Tree SHAP waterfall plot depicting the top 30 predictors of mild cognitive impairment. Predictors are plotted in descending order of model contribution. The bars depict the individual composition ratio (i.e., the amount that each predictor contributes to the classification model; see top of Figure for scale). The curved line represents the cumulative ratio (i.e., the total amount each successive predictor contributes to the model; see bottom of Figure for scale). Leading predictors are depicted above the red line. Abbreviations: QoL, quality of life; hba1c, glycated hemoglobin; BP, blood pressure; meds, medication

The RF classification model demonstrated excellent discrimination of these cohorts (AUC = 0.88; F_1_ score = 0.84; see Table 2 for additional metrics). The Tree SHAP waterfall plot (Fig. 3) shows that the top five predictors explained 51% of the model. They are: QoL (memory; explained 25%), sex (explained 9%), lymphocyte count (explained 8%), self-rated eyesight (explained 5%), and QoL (leisure; explained 4%).

**Fig. 4:**
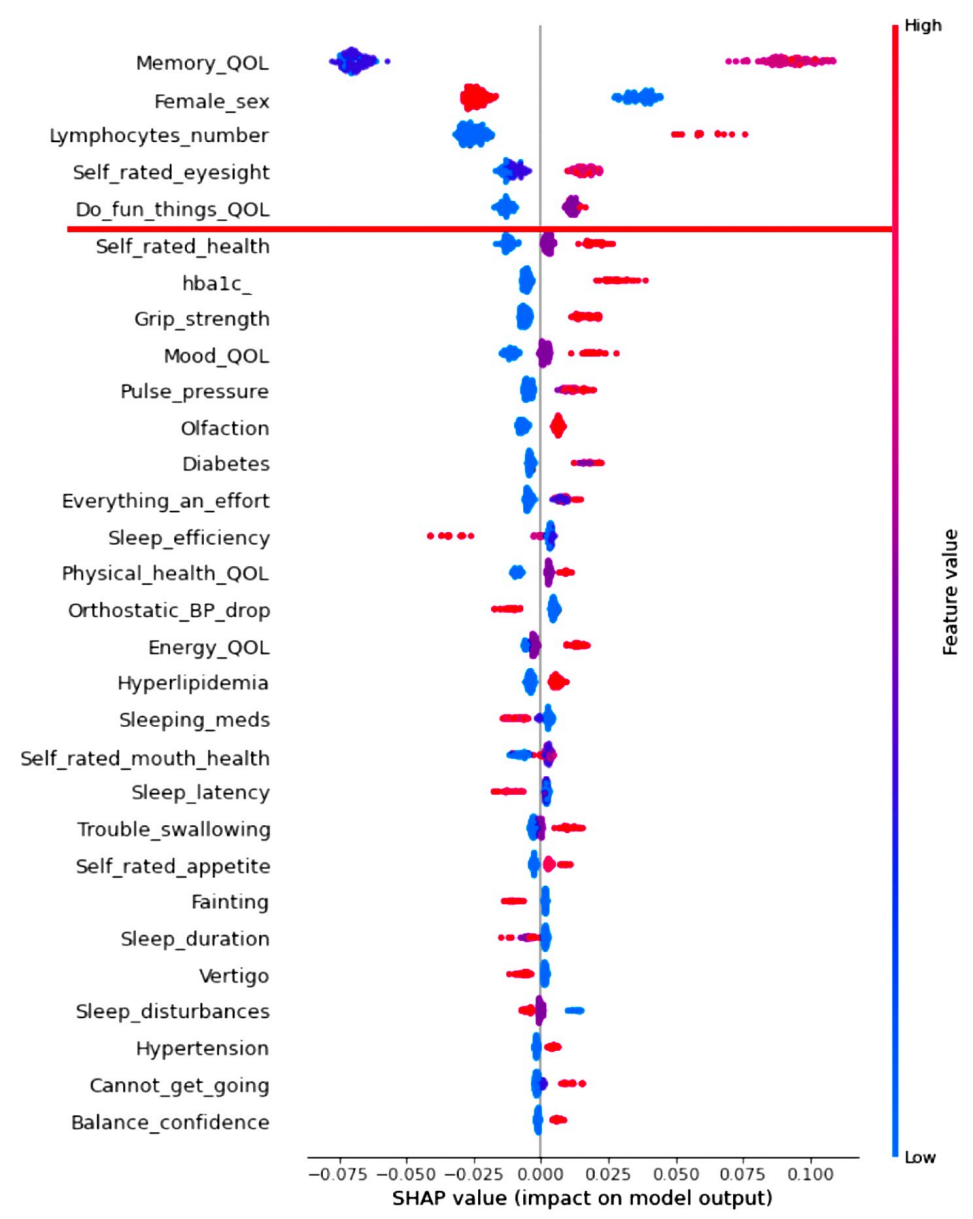
Tree SHAP summary plot depicting the top 30 predictors of mild cognitive impairment. Predictors are plotted in descending order of model contribution. Each dot represents one study participant. These dots collectively represent the direction, prevalence, and magnitude of prediction effects (see Methods section for details). Leading predictors are denoted above the red line. Abbreviations: QoL, quality of life; hba1c, glycated hemoglobin; BP, blood pressure; meds, medication

Regarding direction, the Tree SHAP summary plot (Fig. 4) indicates that increasingly poorer ratings of quality life QoL (memory, leisure), male sex, abnormal lymphocyte count, and higher perceptions of poor self-rated eyesight increase risk for MCI. With respect to prevalence and effect magnitude, for poorer QoL (memory), the large number of red dots along the far-right side of the vertical line indicates that, in general, high-magnitude effects are prevalent. By comparison, male sex is associated with prevalent small-magnitude effects (see densely distributed dots around the lower range). Results for abnormal lymphocyte count reveal that a relatively small proportion of the overall study sample evidenced moderate-to-high effect magnitudes. The pattern of findings for poorer self-rated eyesight and QoL (leisure) are similar, whereby a large number of participants exhibited small magnitude effects.

### RG3: discriminating AD and CU cohorts: machine learning analyses and Tree SHAP interpretation

**Fig. 5:**
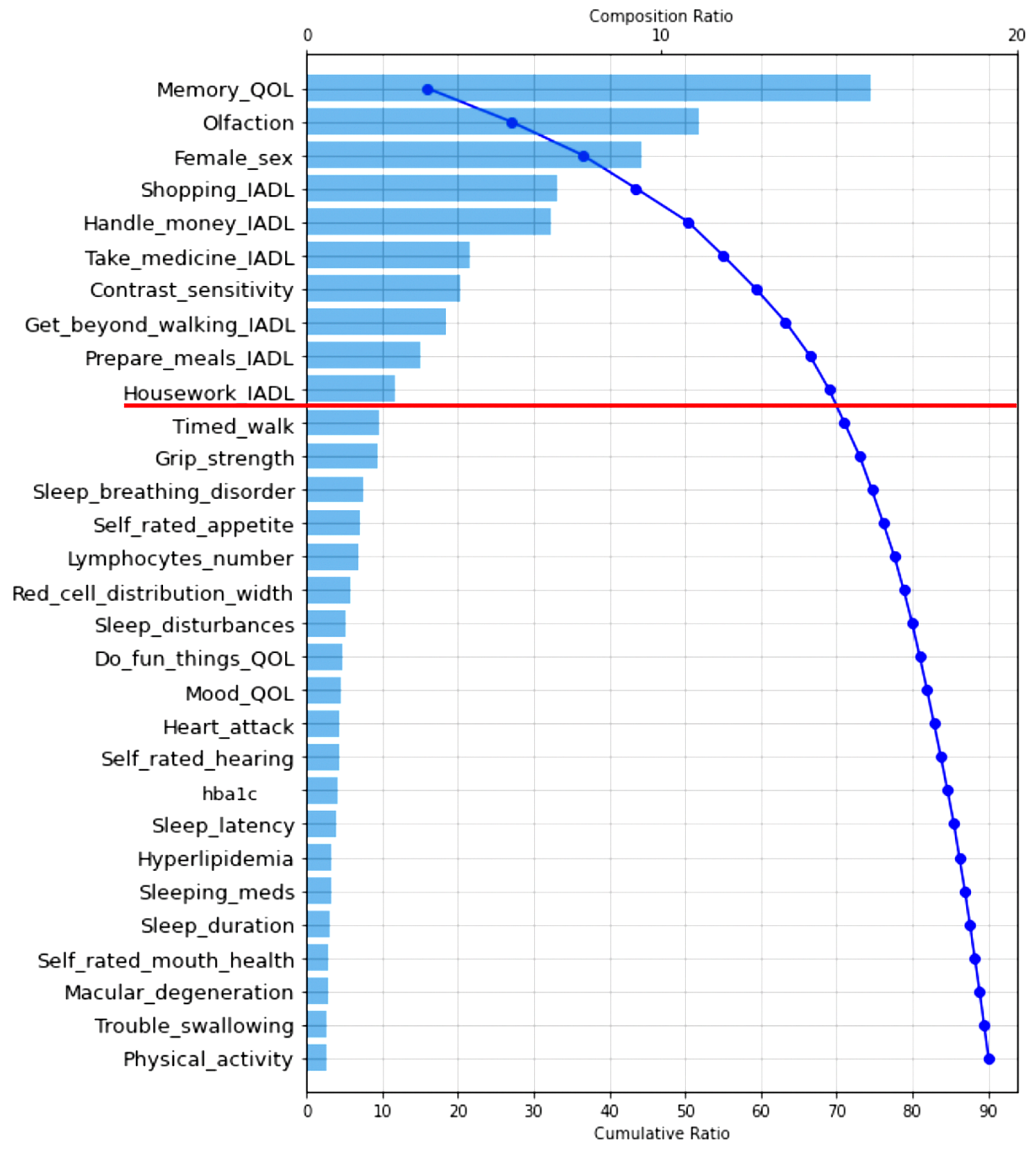
Tree SHAP waterfall plot depicting the top 30 predictors of Alzheimer’s disease. Predictors are plotted in descending order of model contribution. The bars depict the individual composition ratio (i.e., the amount that each predictor contributes to the classification model; see top of Figure for scale). The curved line represents the cumulative ratio (i.e., the total amount each successive predictor contributes to the model; see bottom of Figure for scale). Leading predictors are depicted above the red line. Abbreviations: QoL, quality of life; IADL, instrumental activities of daily living; hba1c, glycated hemoglobin; meds, medication

Evaluation metrics indicated that the RF classification model was characterized by outstanding discrimination of the two cohorts (AUC = 0.98; F_1_ score = 0.84; see Table 2 for additional metrics). As depicted in the Tree SHAP waterfall plot (Fig. 5), the top 10 predictors explained 70% of the model. These include: QoL (memory; explained 15%), olfaction (explained 11%), sex (explained 9%), ability to go shopping (explained 7%), ability to handle money (explained 7%), ability to take medication (explained 5%), visual contrast sensitivity (explained 5%), ability to get to places beyond walking distance (explained 4%), ability to prepare own meals (explained 4%), and ability to do housework (explained 3%).

**Fig. 6:**
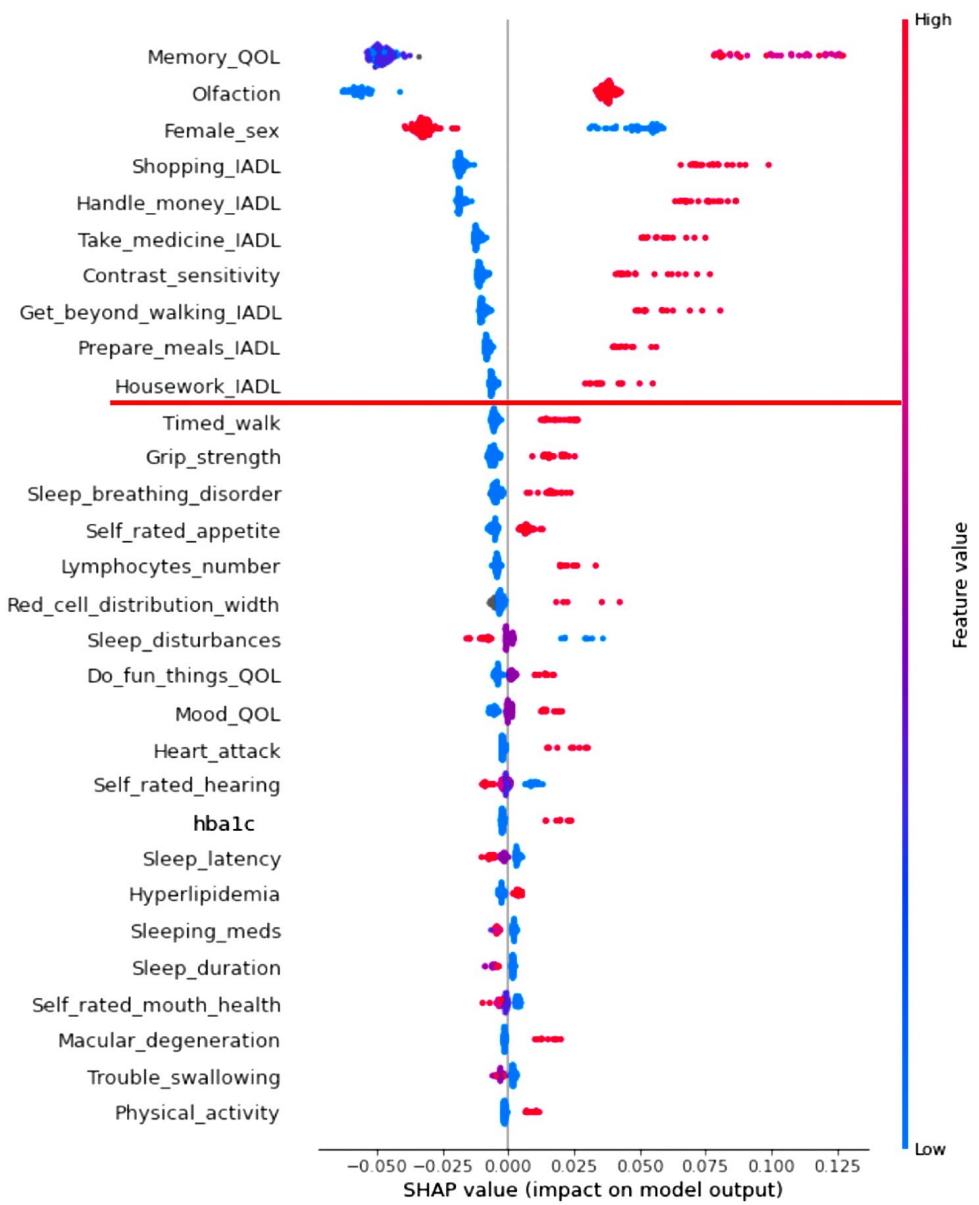
Tree SHAP summary plot depicting the top 30 predictors of Alzheimer’s disease. Predictors are plotted in descending order of model contribution. Each dot represents one study participant. These dots collectively represent the direction, prevalence, and magnitude of prediction effects (see Methods section for details). Leading predictors are denoted above the red line. Abbreviations: QoL, quality of life; IADL, instrumental activities of daily living; hba1c, glycated hemoglobin; meds, medication

Regarding direction, the Tree SHAP summary plot (Fig. 6) reveals that increasingly poorer ratings of QoL (memory), reduced olfaction, male sex, higher levels of dependence in instrumental ADL (*n* indicators = 6), and increasingly poor visual contrast sensitivity increase risk for AD. With respect to prevalence and effect magnitude, poorer QoL (memory), increased dependence in shopping, and increased dependence in handling money were characterized by moderate-to-high magnitude effects for a large number of study participants. Findings for poorer visual contrast sensitivity and increased dependence in taking medication, walking, and preparing meals suggest that, for a large number of study participants, effects are in the moderate range. The observed pattern for olfaction indicates that this predictor is associated with prevalent small-to-moderate effect magnitudes. Effect magnitudes for male sex and increased dependence in housework are also in this range; however, the prevalence of effect magnitudes varies more widely.

## Discussion

We investigate the question of whether constituent factors of frailty may vary in their importance in discriminating CU aging from three clinical conditions in the AD spectrum. Accordingly, we focused not on frailty per se as a general condition predictive of adverse outcomes but on a large pool of constituent factors as potentially dynamic and differentially predictive of specific clinical conditions. The current collection of factors represents standard frailty-related clusters, such as disease syndromes, clinical symptoms, biomarkers, risk factors, medical signals, cognitive characteristics, and health beliefs or practices [13]. Accumulating evidence indicates that progression along fitness-frailty continua predicts an increased risk for exacerbated cognitive decline [71, 72], as well as SCI [2, 3], MCI [4], and AD [5, 6]. We are unaware of prior research that investigated the number and profile of frailty factor signatures that optimize differential prediction of these prominent geriatric conditions.

Our approach applied a combination of ML classifier analysis and explainable artificial intelligence interpretations to a comprehensive database of 83 frailty-related indicators in order to (a) identify the leading factors that discriminate a benchmark CU cohort from SCI, MCI, and AD cohorts and (b) characterize the direction, prevalence, and magnitude of prediction effects. The three RF classifier models demonstrated excellent to outstanding discriminatory ability of these outcomes, with all AUCs in the 0.84–0.98 range. A comparison of the leading predictors extracted across all models (*n* = 14) revealed both convergent (i.e., indicators predictive of two or three cohort memberships; *n* = 3) and cohort-specific (i.e., indicators predictive of only one cohort membership; *n* = 11) factors. Regarding convergence, one indicator, quality of life (memory), discriminated the CU cohort from all three cohorts of the AD spectrum. In addition, the biomarker, lymphocyte count, discriminated the CU cohort from both the SCI and MCI cohorts. Sex (male) discriminated the CU cohort from both the MCI and AD cohorts. Regarding specificity, one indicator selectively predicted SCI (neutrophil count), two indicators predicted only MCI (self-reported eyesight, leisure-related QoL), and eight separate indicators predicted AD (olfaction, visual contrast sensitivity, shopping, handling money, taking medication, getting beyond walking distance, preparing meals, housework). Follow-up Tree SHAP interpretations indicated a generally expected and interpretable direction of effects, whereby higher levels of impairment on the associated indicator predicted an increased risk for clinical cohort membership. We discuss (a) the leading predictors for each clinical cohort and (b) factors that are commonly associated with aging, impairment, and dementia but did not predict any of the geriatric outcome conditions.

### Leading predictors of the SCI cohort

The RF classification model was comprised of 64 multi-modal indicators which collectively contributed to excellent discriminatory ability of the neighboring CU and SCI cohorts (AUC = 0.89; F_1_ score = 0.72). As indicated by the Tree SHAP waterfall plot, the three predictors that most powerfully discriminated the SCI cohort collectively explained nearly half of the model’s fit efficiency (46%). The Tree SHAP summary plot informed the following integrative interpretations.

First, poorer QoL (memory) increased risk for SCI. This study is the first to our knowledge to (a) simultaneously test multiple facets of QoL (memory, physical health, energy, mood, chores, leisure) in a competitive computational context and (b) extract subjective memory perceptions as a critical component that increases SCI risk. Several studies have evaluated the independent association between SCI and QoL (operationalized as life satisfaction, well-being, physical health, or mental health), with results suggesting that poorer perceptions increase risk [73]. The emerging evidence on interventions designed to improve QoL in samples with cognitive impairment or dementia is promising [74–76]. Future studies are therefore encouraged to evaluate whether such interventions may have direct effects on enhancing memory-related QoL.

Second, abnormal lymphocyte and neutrophil counts were leading predictors. The importance of these inflammatory biomarkers in discriminating MCI or AD from CU aging is a topic of increased research interest [77]. To our knowledge, no prior studies have evaluated whether these indicators are also associated with clinical risk for SCI. The present study fills this gap and advances previous ML biomarker research that identified abnormal lymphocyte and neutrophil counts as leading risk characteristics for intensive care unit admission [78], infection [79], and mortality [78, 80]. Pharmacologic or lifestyle interventions targeting systemic inflammation may have positive downstream effects on preventing conversion to MCI or AD, as well as related adverse geriatric outcomes.

### Leading predictors of the MCI cohort

The present model was comprised of 65 multi-modal indicators which produced excellent discrimination of the MCI and CU cohorts (AUC = 0.88; F_1_ score = 0.84). The Tree SHAP waterfall plot revealed that the five leading predictors explained half of the model’s fit efficiency (51%). The Tree SHAP summary plot proffered the following integrative interpretations.

First, poorer memory- and leisure-related QoL were leading predictors. Complementary findings have been reported in related research. For example, informant reports of memory concerns discriminate older adults with cognitive dysfunction from those who are CU [81]. Other research employing RF classifier technologies found that limited activity participation was a top predictor of conversion from CU aging to MCI [79]. Hassler and colleagues [30] reported that reduced activity participation and boredom reliably discriminated frail and non-frail older adults. Although these indicators do not explicitly measure QoL, the general pattern is consistent with the notion that poorer subjective perceptions of memory- and lifestyle-related QoL [82] may be an important indicator of clinical risk for differential objective cognitive decline [10, 16], MCI [17], and AD [18]. These data provide indirect support to recent geriatric research encouraging clinical attention to subjective memory aging, lifestyle, and their potentially broad ramifications on everyday function [73, 74, 83].

Second, male sex increased risk for MCI cohort classifications. This finding buttresses previous work suggesting that— because the prevalence and incidence of MCI is higher amongst males as compared to females [84–86]— males may be more vulnerable to cognitive impairment and dementia [87]. In line with this assertion, Na [79] applied ML analyses to an inventory of sociodemographic, health, interpersonal, quality of life, and well-being indicators and determined that male sex was a key risk signature for MCI. This is a priority area of continued research attention. Advanced understanding of sex differences in risk for impairment and dementia may reveal novel modifiable targets for precision intervention and treatment protocols [88, 89].

Third, abnormal lymphocyte count was a critical discriminative factor. This finding, together with related (non-ML) research, indicates that lymphocytes are a promising MCI biomarker. Specifically, it has been reported that (a) older adults with MCI have higher lymphocytes counts relative to CU samples [77] and (b) these cohorts are reliably discriminated by this routine parameter [90, 91]. We clarify and extend this prior work by (a) applying ML technologies to multiple frailty-related risk modalities and (b) extracting lymphocytes as a critical predictor of subjective and objective cognitive impairment. The mechanisms underlying these associations are not yet well understood [77, 91]. However, it is possible that sustaining abnormal levels of this pro-inflammatory cytokine may contribute to adverse changes in brain structure and function (e.g., interrupt adult neurogenesis) [92].

Fourth, poorer self-rated eyesight was a leading predictor. Much of the available geriatric research evaluating vision-MCI associations has operationalized impairment using objective measures of distance visual acuity [93], with results suggesting that such factors increase MCI risk [94]. Because visual function is a complex process, it has been suggested that future investigations should evaluate a wider breadth of visual function indicators [95]. The current study addressed this research direction by testing the relative predictive importance of both objective (contrast sensitivity, macular degeneration, cataracts) and subjective (self-reported eyesight) indicators in a computationally competitive context. Our results suggest that routine monitoring of self-reported eyesight may promote earlier identification of at-risk older adults in clinical care settings.

### Leading predictors of the AD cohort

The current model was comprised of 75 multi-system indicators which collectively contributed to outstanding discrimination of the two cohorts (AUC = 0.98; F_1_ score = 0.84). Evidence depicted in the Tree SHAP waterfall plot revealed that the 10 leading predictors explained 70% of the model’s fit efficiency. As discussed above, the Tree SHAP summary plot indicated that poorer QoL (memory) was a critical factor discriminating the AD and CU cohorts. Integrative interpretations for the remaining predictors are provided below.

First, two sensory factors, reduced olfaction and poorer visual contrast sensitivity, predicted AD. Several studies have indicated a need for epidemiological research to evaluate the relative importance of olfaction, vision, and audition in predicting cognitive impairment and AD [20, 96–98]. Our sensory-related findings showed that olfaction was the leading risk domain, followed by vision. Notably, neither of the audition indicators (self-reported hearing, objective hearing impairment) were amongst the critical predictors of AD. The rank-ordering of these sensory risk factors replicates related (non-ML) geriatric research examining the relative importance of olfaction, vision, and audition in predicting accelerated cognitive decline and MCI [96, 97].

Second, convergent with MCI-related results, male sex was a leading predictor of AD. These data suggest that male sex may serve as a proxy for generalized morbidity or deficit accumulation in aging [99]. Future replications could evaluate whether this pattern may also be attributed to the unbalanced sex distributions across the CU (18% male), MCI (51% male), and AD (70% male) cohorts.

Third, each of the instrumental ADL indicators tested in the present model emerged as salient discriminative factors. These included increased dependence in shopping, handling money, taking medication, getting to places beyond walking distance, preparing meals, and performing housework. Complementary findings were reported in a recent study evaluating the independent association between instrumental ADL and AD-related neurodegeneration biomarkers [100], whereby increased difficulties in shopping, balancing a check book, and managing medications were correlated with smaller hippocampal volumes and reduced brain network connections. Previous ML work evaluated the relative predictive importance of instrumental ADL indicators and related modalities in discriminating frail and non-frail older adults. Leading predictors included reduced kitchen activity levels and kitchen use duration [101], as well as increased difficulties in shopping, cooking, performing housework, and using public transportation or the telephone [30]. Instrumental ADL summary scores have also been shown to (a) discriminate AD and CU cohorts [102, 103] and (b) predict risk of converting from SCI [104] or MCI [105] to AD. The latter findings suggest that deficits in this domain may appear early in the neuropathological cascade of AD [106, 107]. However, we did not detect such a pattern in our results. It is possible that older adults with SCI or MCI may be more likely to demonstrate reduced instrumental ADL capabilities at more advanced ages or at higher levels of perceived or objective impairment. Alternatively, participants in each of the respective cohorts may represent a relatively high functioning subset of older adults due to the demanding nature of the COMPASS-ND study protocol [36, 41].

### What about the trailing predictors (falling below the break in the Tree SHAP waterfall plots)?

Our discussion concentrates on factors identified as among the leading predictors of the three AD-related cohorts. However, all of the indicators included in the prediction models have been previously identified as potential contributors to geriatric frailty in aging and dementia. In the present computationally competitive RF classification models, many of these factors did not appear as relatively important predictors for any clinical cohort. As depicted in the Tree SHAP waterfall plots: (a) the top 30 predictors collectively explained approximately 90% of each model’s fit efficiency (range = 86–91%); (b) this variance was largely accounted for by the leading predictors (i.e., those above the break in the cumulative ratio; range = 46–70%); and (c) the remaining predictors explained a comparatively smaller proportion the model’s fit efficiency (range = 20–40%). In Supplement Table 2, we (a) present a complete list of indicators that were tested in the three classifier models and (b) denote indicators that were not robust predictors of any cohort in the AD spectrum. As can be seen, none of the indicators from the following modalities were extracted in the present computational models: basic ADL, physical activity, mobility, anthropometric measures, sleep, functional indicators, exhaustion, self-reported health, cardiorespiratory health, clinical symptoms and diseases, emotional wellbeing, and oral health or nutritional factors. Each of these domains have been characterized (to varying degrees) as important risk elevating characteristics for cognitive impairment and dementia [98, 108]. However, our results suggest that, when simultaneously analyzed in a powerful ML context, these facets of frailty are comparatively less important than QoL, fluid biomarkers, sensory function, sex, and instrumental ADL.

### Strengths and limitations

We note the following study strengths. First, we applied a combination of binary RF classifier analysis and Tree SHAP interpretations to a broad swath of frailty factors. The integration of ML and explainable artificial intelligence technologies represents a promising complement to traditional research methods, such as candidate factor or composite index approaches. Supplemental advantages include the opportunity for (a) extracting critical predictors of SCI, MCI, and AD from a large cluster of frailty-related morbidity, deficit, and risk indicators; (b) identifying clinically relevant and potentially unexpected or unique signatures of frailty and dementia risk; and (c) elucidating the direction, prevalence, and magnitude of prediction effects while at the same time controlling for overfitting [25, 58, 59]. Future studies are encouraged to examine concordant research questions using complementary ML algorithms and approaches (e.g., discrimination of SCI, MCI, and AD cohorts using multi-class RF analysis). Second, each of the three RF models were characterized by a low proportion of missing data (1–2%). We estimated these values using a sophisticated [66] and accurate [45] imputation approach, as applied in the *sklearn* pipeline [51]. Relative to listwise or pairwise deletion, multiple imputation approaches are associated with reduced bias and increased generalizability of the study findings [47]. Third, cross-sectional data were drawn from the COMPASS-ND database, which represents a comprehensive Canadian database of geriatric neurodegenerative disorders [36, 37]. Participants were deeply phenotyped cohorts who collectively represented the full AD spectrum. Inclusion of CU older adults allowed us to detect potentially modifiable risk characteristics that could be targeted prior to the onset of clinically detectable frailty, cognitive impairment, or dementia.

We note the following study limitations. First, participants in our study were primarily non-Hispanic White (92%), compromising generalizability to older adults of diverse racial, ethnic, or indigenous backgrounds. Second, the present sample was community dwelling, perhaps suggesting a generally more functional lifestyle than would be expected for persons living with the advanced clinical conditions in formal care settings. Additional research reflecting diverse lifestyle and social living settings is encouraged. Third, because the COMPASS-ND dataset is unbalanced by sex, we were unable to examine disaggregated models. However, testing for sex as a factor revealed that in two (of the three) models, sex was a leading predictor. Further research on sex and gender contributions is recommended. Fourth, slightly different subsets of the original 83 identified frailty-related indicators were evaluated in the three RF classification models. As noted in the Methods section, some indicators were differentially available across the cohorts due to (a) missing data that exceeded the criterion cut-off of 50% [34, 45–47] or (b) a low proportion of participants (< 10%) characterized by the associated deficit [35, 48]. See Supplement Table 2 for a complete description. Follow-up studies could explore whether complementary prediction patterns are detected when such indicators are uniformly available.

## Conclusions

The current study tested a wide and representative set of up to 75 frailty-related factors (overall pool *n* = 83) frequently used in geriatric research. We used three computationally competitive ML models to identify salient predictors that discriminate a cohort of CU older adults from those with SCI, MCI, or AD. Examination of the leading predictors extracted across the three classifier models suggested both convergence and specificity of deficit-related prediction patterns. Regarding convergence: (a) QoL (memory) predicted each phase of the AD spectrum, such that older adults characterized by perceived or objective impairment found that poor memory affected their quality of life; (b) abnormal lymphocyte count predicted both SCI and MCI; and (c) male sex predicted both MCI and AD. A morbidity intensity trend was indicated by an increasing number and diversity of predictors corresponding to clinical severity. Specifically: (a) SCI was selectively predicted by abnormal neutrophil count; (b) MCI was selectively predicted by poorer self-reported eyesight and leisure-related QoL; and (c) AD was selectively predicted by reduced olfaction, poorer visual contrast sensitivity, and increased dependence in instrumental ADL (shopping, handling money, taking medication, getting beyond walking distance, preparing meals, housework). These results advance precision understanding of morbidity and deficit accumulation and its impact across the AD spectrum. Importantly, the majority of these factors are potentially modifiable and may therefore include potential targets for early interventions designed to offset or delay the incidence of cognitive impairment or dementia [109]. Collectively, this knowledge may better enable precision health solutions to identify and target signatures of risk as specific phases of the AD spectrum. Future longitudinal extensions in the COMPASS-ND database and related large-scale geriatric studies are encouraged.

### Electronic supplementary material

Below is the link to the electronic supplementary material.

Supplementary Material 1

## Data Availability

The authors will make data available upon reasonable request to the corresponding author and approval by the Canadian Consortium on Neurodegeneration in Aging Publications and Data Access Committee.

## Abbreviations

AD: Alzheimer’s disease
ADL: Activities of daily living
AUC: Area under the receiver operating characteristic curve
CCNA: Canadian Consortium on Neurodegeneration in Aging
COMPASS-ND: Comprehensive Assessment of Neurodegeneration and Dementia
CU: Cognitively unimpaired
MCI: Mild cognitive impairment
ML: Machine learning
QoL: Quality of life
RF: Random forest
RG: Research goals
SCI: Subjective cognitive impairment
Tree SHAP: Tree Shapley Additive exPlanation values
