# Supplementary material for: Machine learning analyses identify multi-modal frailty factors that selectively discriminate four cohorts in the Alzheimer’s disease spectrum: a COMPASS-ND study
Source: BMC Geriatr. 2023 Dec 11;23:837. doi: 10.1186/s12877-023-04546-1 (PMC10714519; doi:10.1186/s12877-023-04546-1)
Supplement: Supplementary file 1 — Supplementary Material 1 [file 12877_2023_4546_MOESM1_ESM.docx]

**Additional File 1:** Bohn et al. Machine Learning Analyses Identify Multi-Modal Frailty Factors that Selectively Discriminate Four Cohorts in the Alzheimer’s Disease Spectrum: A COMPASS-ND Study

**Supplement Table 1** Response Scales for Frailty-Related Indicators Eligible for Inclusion in Random Forest Analysis (*n* = 83)

| **Domain** | **Indicator** | | | | **Response Scale** |  |
| --- | --- | --- | --- | --- | --- | --- |
| **IADL** | Getting to places beyond walking distance | | 0 = without help; 0.5 = with some help; 1 = completely unable | | |  |
|  | Going shopping for groceries or clothes | |  |  |  |  |
|  | Preparing meals | |  |  |  |  |
|  | Doing housework | |  |  |  |  |
|  | Taking medication | |  | | |  |
|  | Handling money | |  | | |  |
| **ADL** | Trouble getting to bathroom in time | | 0 = no; 1 = yes | | |  |
| **Physical activity** | Physical activity (PASE score) | | ﻿0 = ≥ 64 males; ≥ 52 females  1 = < 64 males; < 52 females | | |  |
| **Mobility** | Self-reported balance | | 0 = very good; 0.5 = pretty good; 1 = very poor | | |  |
|  | Balance confidence (ABC score) | | 0 = high; 0.5 = moderate; 1 = low | | |  |
|  | Timed walk (averaged over three 6m trials) | | 0 = > 1m/s;1 = < 1m/s | | |  |
|  | Falls in the past year | | 0 = no; 1 = yes | | |  |
| **QoL** | Physical health | | 0 = excellent; 0.33 good; 0.66 = fair; 1 = poor | | |  |
|  | Energy | |  |  |  |  |
|  | Mood | |  | | |  |
|  | Memory | |  | | |  |
|  | Ability to do chores around the house | |  | | |  |
|  | Ability to do things for fun | |  | | |  |
| **Anthropometric measures** | Waist-to-hip ratio | | 0 = < 0.85 females; < 0.96 males 1 = > 0.85 females, > 0.96 males | | |  |
|  | Waist circumference (cm) | | 0 < 88 females; < 102 males  1 > 88 females; > 102 males | | |  |
|  | Body mass index (kg/m^2^) | | 0 = 18.5-25; 0.5 = 25 to < 30;  1 = < 18.5 or ≥ 30 | | |  |
|  | Unintentional weight loss | | 0 = no; 1 = yes | | |  |
| **Sensory function** | Self-reported eyesight | | 0 = excellent; 0.25 = very good; 0.50 = good; 0.75 = fair; 1 = poor or non-existent | | |  |
|  | Self-reported hearing | |  |  |  |  |
|  | Hearing handicap (HHIE score) | | 0 = no hearing handicap; 0.5 = mild/moderate handicap; 1 = significant handicap | | |  |
|  | Olfaction (B-SIT score) | | 0 = > 11; 1 = < 10 | | |  |
|  | Visual contrast sensitivity (Mars Letter Contrast Sensitivity Test) | | 0 = normal; 0.5 = moderate; 1 = severe impairment | | |  |
|  | Cataracts | | 0 = no; 1 = yes | | |  |
|  | Macular degeneration | | 0 = no; 1 = yes | | |  |
| **Sleep** | Sleep duration (PSQI score) | | 0 = none; 0.33 = slight problem; 0.66 = somewhat a problem; 1 = big problem | | |  |
|  | Sleep efficiency (PSQI score) | |  |  |  |  |
|  | Sleep disturbances (PSQI score) | |  |  |  |  |
|  | Daytime dysfunction (PSQI score) | |  |  |  |  |
|  | Sleep latency (PSQI score) | |  |  |  |  |
|  | Sleep medication (PSQI score) | |  |  |  |  |
|  | Self-reported sleep quality | | 0 = very good; 0.33 = fairly good; 0.66 = fairly bad; 1 = very bad | | |  |
| **Functional indicators** | Grip strength (averaged over three trials) | | Males: for BMI ≤ 24, GS ≤ 29; for BMI 24.1-28, GS ≤ 30; for BMI > 28, GS ≤ 32  Females: for BMI ≤ 23, GS ≤ 17; for BMI 23.1-26, GS ≤ 17.3; for BMI 26.1-29, GS ≤ 18; for BMI > 29, GS ≤ 21 | | |  |
|  | Pulse pressure (mmHg) | | 0 = 32.13-63.90; 0.5 = 64-75.9; 1 = < 32.12 or 76+ | | |  |
|  | Resting heart rate (bpm) | | 0 = 60-99; 1 = < 60 or 100 < | | |  |
| **Exhaustion** | Everything an effort  Could not get going | | 0 = rarely or none of the time; 0.25 = some or a little of the time; 0.75 = a moderate amount; 1 = most of the time | | |  |
| **Self-reported health** | | Current health | | 0 = very good; 0.25 = good; 0.50 = average; 0.75 = poor; 1 = very poor | | |
| **Cardiorespiratory health** | Chronic respiratory condition | | 0 = no; 1 = yes | | |  |
|  | Shortness of breath | |  | | |  |
|  | Sleep breathing disorder | |  | | |  |
|  | High blood pressure or hypertension | |  | | |  |
|  | ﻿Atrial fibrillation or irregular heartbeat | |  | | |  |
|  | ﻿Heart attack, congestive heart failure | |  | | |  |
|  | Peripheral vascular disease | |  | | |  |
|  | Mini-stroke or TIA | |  | | |  |
|  | Hyperlipidemia | |  | | |  |
| **Clinical symptoms or diseases** | Episodes of fainting | | 0 = no; 1 = yes | | |  |
|  | Orthostatic blood pressure drop | |  | | |  |
|  | Vertigo or dizziness | |  | | |  |
|  | Type II diabetes | | 0 = no; 0.5 = borderline/high blood sugar; 1 = type I or II diabetes | | |  |
|  | Polypharmacy | | 0 = 0-4 medications; 0.5 = 5-7 medications; 1 = 8+ medications; 2 = 14+ medications | | |  |
|  | Osteoarthritis | | 0 = no; 1 = yes | | |  |
|  | Hypothyroidism | | 0 = no; 1 = yes | | |  |
|  | Osteoporosis | | 0 = no; 1 = yes | | |  |
|  | Stomach ulcers | | 0 = no; 1 = yes | | |  |
|  | Irritable bowel syndrome | | 0 = no; 1 = yes | | |  |
|  | Chronic constipation | | 0 = no; 1 = yes | | |  |
|  | Urinary incontinence | | 0 = no; 1 = yes | | |  |
|  | Cancer | | 0 = no; 1 = yes | | |  |
| **Emotional wellbeing** | Major depressive disorder | | 0 = no; 1 = yes | | |  |
|  | Generalized anxiety disorder | |  | | |  |
| **Oral health and nutritional factors** | Self-reported appetite | | 0 = very good; 0.33 = good; 0.66 = fair; 1 = poor | | |  |
|  | Coughing, choking, pain when swallowing | | 0 = never; 0.33 = rarely; 0.66 = sometimes; 1 = often or always | | |  |
|  | Self-reported mouth health | | 0 = excellent; 0.25 = very good; 0.50 = good; 0.75 = fair; 1 = poor or non-existent | | |  |
|  | Eating discomfort due to mouth problems | | 0 = never; 0.33 = rarely; 0.66 = sometimes; 1 = often or always | | |  |
|  | Avoid eating particular food due to mouth | |  | | |  |
| **Fluid biomarkers** | Hemoglobin | | 0 = inside established reference range; 1 = outside established reference range | | |  |
|  | HbA1c | |  |  |  |  |
|  | Mean corpuscular hemoglobin concentration | |  |  |  |  |
|  | Mean corpuscular hemoglobin | |  |  |  |  |
|  | Mean corpuscular volume | |  | | |  |
|  | White blood cell count | |  | | |  |
|  | Red blood cell count | |  | | |  |
|  | Red cell distribution width | |  | | |  |
|  | Number of lymphocytes | |  | | |  |
|  | Number of neutrophils | |  | | |  |
|  | Hematocrit | |  | | |  |
| **Sex** | Male or Female | | 0 = male; 1 = female | | |  |

Abbreviations: IADL, instrumental activities of daily living; ADL, basic activities of daily living; QoL, quality of life; PASE, adapted Physical Activity Scale for the Elderly [1]; ABC, Activities-specific Balance Confidence Scale [2]; HHIE, Hearing Handicap Inventory for the Elderly— screening version [3]; B-SIT, Brief Smell Identification Test [4]; PSQI, Pittsburgh Sleep Quality Index [5]; TIA, transient ischemic attack; HbA1c, glycated hemoglobin.

**Supplement Table 2** Predictors Below the Break in the Tree SHAP Waterfall Plots

| **Indicator** | **SCI** | **MCI** | **AD** |
| --- | --- | --- | --- |
| Getting to places beyond walking distance (IADL) | ^^^ | ^^^ | ^*^ |
| Going shopping for groceries or clothes (IADL) | ^^^ | ^^^ | ^*^ |
| Preparing own meals (IADL) | ^^^ | ^^^ | ^*^ |
| Doing housework (IADL) | ^X^ | ^^^ | ^*^ |
| Taking own medication (IADL) | ^^^ | ^^^ | ^*^ |
| Handling own money (IADL) | ^^^ | ^^^ | ^*^ |
| Trouble getting to bathroom in time (ADL) | ^X^ | ^X^ | ^X^ |
| Physical activity | ^X^ | ^X^ | ^X^ |
| Self-reported balance | ^X^ | ^X^ | ^X^ |
| Balance confidence | ^X^ | ^X^ | ^X^ |
| Timed walk | ^X^ | ^X^ | ^X^ |
| Falls in the past year | ^X^ | ^X^ | ^X^ |
| Physical health QoL | ^X^ | ^X^ | ^X^ |
| Energy QoL | ^X^ | ^X^ | ^X^ |
| Mood QoL | ^X^ | ^X^ | ^X^ |
| Memory QoL | ^*^ | ^*^ | ^*^ |
| Ability to do chores around the house QoL | ^X^ | ^X^ | ^X^ |
| Ability to do things for fun QoL | ^X^ | ^*^ | ^X^ |
| Waist-to-hip ratio | ^X^ | ^X^ | ^X^ |
| Waist circumference | ^X^ | ^X^ | ^X^ |
| Body mass index | ^X^ | ^X^ | ^X^ |
| Unintentional weight loss | ^X^ | ^^^ | ^X^ |
| Self-reported eyesight | ^X^ | ^*^ | ^X^ |
| Self-reported hearing | ^X^ | ^X^ | ^X^ |
| Hearing handicap | ^X^ | ^X^ | ^X^ |
| Olfaction | ^X^ | ^X^ | ^*^ |
| Visual contrast sensitivity | ^^^ | ^X^ | ^*^ |
| Cataracts | ^X^ | ^X^ | ^X^ |
| Macular degeneration | ^^^ | ^^^ | ^X^ |
| Sleep duration (PSQI score) | ^X^ | ^X^ | ^X^ |
| Sleep efficiency (PSQI score) | ^X^ | ^X^ | ^X^ |
| Sleep disturbances (PSQI score) | ^X^ | ^X^ | ^X^ |
| Daytime dysfunction (PSQI score) | ^X^ | ^X^ | ^X^ |
| Sleep latency (PSQI score) | ^X^ | ^X^ | ^X^ |
| Sleep medication (PSQI score) | ^X^ | ^X^ | ^X^ |
| Self-reported sleep quality | ^X^ | ^X^ | ^X^ |
| Grip strength | ^X^ | ^X^ | ^X^ |
| Pulse pressure | ^X^ | ^X^ | ^X^ |
| Resting heart rate | ^X^ | ^X^ | ^X^ |
| Fatigue- everything an effort | ^X^ | ^X^ | ^X^ |
| Fatigue- could not get going | ^X^ | ^X^ | ^X^ |
| Self-reported current health | ^X^ | ^X^ | ^X^ |
| Self-reported appetite | ^X^ | ^X^ | ^X^ |
| Coughing, choking, pain when swallowing | ^X^ | ^X^ | ^X^ |
| Self-reported mouth health | ^X^ | ^X^ | ^X^ |
| Eating discomfort due to mouth problems | ^X^ | ^X^ | ^X^ |
| Avoid eating particular food due to mouth | ^X^ | ^X^ | ^X^ |
| Polypharmacy | ^X^ | ^X^ | ^X^ |
| Osteoarthritis | ^X^ | ^X^ | ^X^ |
| Chronic respiratory condition | ^X^ | ^X^ | ^X^ |
| Sleep breathing disorder | ^X^ | ^X^ | ^X^ |
| High blood pressure or hypertension | ^X^ | ^X^ | ^X^ |
| Hyperlipidemia | ^X^ | ^X^ | ^X^ |
| Atrial fibrillation or irregular heartbeat | ^X^ | ^X^ | ^X^ |
| Heart attack, congestive heart failure | ^^^ | ^^^ | ^X^ |
| Peripheral vascular disease | ^X^ | ^^^ | ^^^ |
| Mini-stroke or TIA | ^^^ | ^X^ | ^^^ |
| Episodes of fainting | ^X^ | ^X^ | ^X^ |
| Orthostatic blood pressure drop | ^X^ | ^X^ | ^X^ |
| Vertigo or dizziness | ^X^ | ^X^ | ^X^ |
| Type II diabetes | ^^^ | ^X^ | ^X^ |
| Hypothyroidism | ^X^ | ^X^ | ^X^ |
| Osteoporosis | ^X^ | ^X^ | ^X^ |
| Stomach ulcers | ^X^ | ^X^ | ^X^ |
| Irritable bowel syndrome | ^X^ | ^^^ | ^^^ |
| Chronic constipation | ^^^ | ^X^ | ^^^ |
| Urinary incontinence | ^X^ | ^X^ | ^X^ |
| Cancer | ^X^ | ^X^ | ^X^ |
| Major depressive disorder | ^X^ | ^X^ | ^X^ |
| Generalized anxiety disorder | ^X^ | ^X^ | ^X^ |
| Sex | ^X^ | ^*^ | ^*^ |
| Hemoglobin | ^^^ | ^X^ | ^X^ |
| HbA1c | ^X^ | ^X^ | ^X^ |
| Mean corpuscular hemoglobin concentration | ^^^ | ^^^ | ^X^ |
| Mean corpuscular hemoglobin | ^^^ | ^^^ | ^X^ |
| Mean corpuscular volume | ^^^ | ^X^ | ^^^ |
| White cell count | ^X^ | ^^^ | ^^^ |
| Red cell count | ^X^ | ^X^ | ^X^ |
| Red cell distribution width | ^^^ | ^^^ | ^X^ |
| Number of lymphocytes | ^*^ | ^*^ | ^X^ |
| Number of neutrophils | ^*^ | ^^^ | ^^^ |
| Hematocrit | ^^^ | ^X^ | ^X^ |

^^^ Denotes indicators that were excluded from the associated pairwise comparison (for reasons as noted in the Methods section). ^X^ Denotes indicators that were below the break in the cumulative ratio (as depicted in the Tree SHAP waterfall plots). ^*^ Denotes indicators that were above the break in the cumulative ratio (as depicted in the Tree SHAP waterfall plots). Abbreviations: RF, random forest; SCI, subjective cognitive impairment; MCI, mild cognitive impairment; AD, Alzheimer’s disease; IADL, instrumental activities of daily living; ADL, basic activities of daily living; QoL, quality of life; PSQI, Pittsburgh Sleep Quality Index; TIA, transient ischemic attack; HbA1c, glycated hemoglobin.

**Supplement References**

1. Washburn RA, Smith KW, Jette AM, Janney CA. The Physical Activity Scale for the Elderly (PASE): Development and evaluation. J Clin Epidemiol. 1993;46(2):153–62.
2. Powell LE, Myers AM. The Activities-specific Balance Confidence (ABC) Scale. Journal of Gerontology Medical Sciences. 1995;50A(1):M28–34.
3. Ventry IM, Weinstein BE. The Hearing Handicap Inventory for the Elderly: A new tool. Ear Hear. 1982;3(3):128–34.
4. Menon C, Westervelt HJ, Jahn DR, Dressel JA, O’Bryant SE. Normative performance on the Brief Smell Identification Test (BSIT) in a multi-ethnic bilingual cohort: A Project FRONTIER study. Clin Neuropsychol. 2013;27(6):946–61.
5. Buysse DJ, Reynolds CF, Monk TH, Berman SR, Kupfer DJ. The Pittsburgh Sleep Quality Index: A new instrument for psychiatric practice and research. Psychiatry Res. 1989;28(2):183–213.
